# PRELIMINARY SOMMA DATA: MITOCHONDRIAL ENERGETICS, LEG POWER, AND CARDIORESPIRATORY FITNESS

**DOI:** 10.1093/geroni/igac059.833

**Published:** 2022-12-20

**Authors:** Theresa Mau, Paul Coen, Stephen Kritchevsky, Anne Newman, Giovanna Distefano, Russell T Hepple, Bret Goodpaster, Steven Cummings

**Affiliations:** California Pacific Medical Center, San Francisco, California, United States; AdventHealth, Orlando, Florida, United States; Wake Forest School of Medicine, Winston-Salem, North Carolina, United States; University of Pittsburgh, Pittsburgh, Pennsylvania, United States; Advent Health Translational Research Institute, Orlando, Florida, United States; University of Florida, Gainesville, Florida, United States; AdventHealth, Orlando, Florida, United States; San Francisco Coordinating Center, San Francisco, California, United States

## Abstract

To assess mitochondrial function, max OXPHOS (maximal complex I and II supported state 3 respiration) was determined in vitro by high-resolution respirometry of permeabilized muscle fibers from thigh muscle (vastus lateralis) biopsies, and ATPmax was determined in vivo by 31P magnetic resonance spectroscopy (n=468, mean=76.9yr). Max OXPHOS was strongly associated with leg power (β= 10.7 Watts per 1 SD increment, p<0.001) and peak VO2 (β= 83.7 mL/min, p<0.001). ATPmax had weak associations with leg power (β= 4.2 Watts per SD increment, p=0.13). In contrast to its weaker relationship with leg power, ATPmax was strongly associated with VO2 peak (β= 70.1 mL/min per SD increment, p<0.001). Skeletal muscle mitochondrial energetics were significant determinants of leg power and VO2 peak in older adults.

